# Gaps in public health surveillance of elective surgery: a national analysis of inpatient data, COVID-19 impact, and coding validity in Germany (2006–2023)

**DOI:** 10.3389/fpubh.2026.1813408

**Published:** 2026-06-17

**Authors:** Maximilian Jacobi, Steffen Roßlenbroich, Jana Roßlenbroich, Maximilian Kueckelhaus, Nadine Giebeler, Tobias Hirsch, Hinne Rakhorst, Sascha Wellenbrock

**Affiliations:** 1 Department of Plastic Surgery, University Hospital Muenster, Muenster, Germany; 2 Department of Plastic and Reconstructive Surgery, Institute for Musculoskeletal Medicine, University of Muenster, Muenster, Germany; 3 Department of Plastic, Reconstructive and Aesthetic Surgery, Hand Surgery, Fachklinik Hornheide, Muenster, Germany; 4 Department of Plastic Surgery, University Medical Center Groningen and University of Groningen, Groningen, Netherlands; 5 Department of Trauma, Hand and Reconstructive Surgery, University Hospital Muenster (UKM), Muenster, Germany; 6 Niels-Stensen Clinics, St. Mary's Hospital Osnabrueck, Osnabrueck, Germany; 7 Reimbursement Institute, Huerth, Germany

**Keywords:** administrative datasets, aesthetic surgery, data validity, patient safety, public health surveillance

## Abstract

**Background:**

Administrative inpatient datasets are cornerstone tools for health surveillance, yet their validity in tracking elective, self-pay surgical markets remain poorly defined. This study evaluates the utility of nationwide inpatient data for monitoring aesthetic surgery by quantifying 18-year trends, assessing the COVID-19 pandemic's impact, and benchmarking hospital capture rates against national estimates to identify critical public health surveillance gaps.

**Methods:**

We conducted a descriptive longitudinal epidemiologic analysis of mandatory German hospital quality reports (2006–2023). Four high-volume procedures (liposuction, blepharoplasty, breast augmentation, and abdominoplasty) were identified via procedure-specific codes. To evaluate diagnostic validity, we analyzed the usage of ICD-10 code Z41.1 (“Procedures for purposes other than remedying health states”). Inpatient volumes were benchmarked against ISAPS national estimates to determine the “hospital share” of the total market.

**Results:**

Between 2006 and 2023, 352,468 inpatient procedures were recorded. Annual volumes tripled from 11,593 to 33,170. While inpatient liposuction (+611%) and abdominoplasty (+424%) surged, breast augmentation declined by 33%. Liposuction volumes showed no significant immediate 2020 level decline in segmented analysis and continued to increase during the post-pandemic period. Benchmarking suggested limited hospital visibility of the broader national aesthetic-surgery market: by 2023, hospitals captured only 2.0% of national breast augmentations and 13.5% of liposuctions. ICD-10 Z41.1 was utilized in only 0.4% of cases, suggesting limited utility as an indication-specific surveillance marker.

**Conclusions:**

While hospital data serve as a vital sentinel for high-complexity cases, the massive discrepancy between inpatient volumes and national estimates highlights a large data gap in elective surgery surveillance. The limited use of Z41.1 suggests that current diagnostic coding frameworks are insufficient, on their own, for monitoring elective self-pay procedures. Transitioning to integrated, cross-sectoral registries and ICD-11 is essential for accurate epidemiological mapping and surgical workforce planning.

## Introduction

Aesthetic surgery has evolved from a niche medical subspecialty into a significant component of elective healthcare global markets. This growth is driven by increasing disposable incomes, the destigmatization of cosmetic interventions, and the proliferation of digital media, which has accelerated the “medicalization” of physical appearance (1, 2). While often viewed through a purely clinical or cosmetic lens, the rapid expansion of this sector presents unique challenges for public health surveillance, patient safety monitoring, and healthcare resource allocation (3).

The public and professional perception of plastic surgery is heavily skewed toward its aesthetic components. Analysis of private-sector healthcare providers in the D-A-CH region (i.e. Germany, Austria and Switzerland) demonstrates that nearly 99% of digital outreach focuses on aesthetic procedures, often at the expense of reconstructive or burn care (1). This commercial visibility has led to a significant “identity gap” within the medical field; surveys indicate that laypeople, medical students, and even primary care physicians frequently equate the entirety of the specialty with elective cosmetic treatment (4–6). In Germany specifically, younger demographics are increasingly likely to view the field solely as an aesthetic consumer service rather than a vital medical specialty, a perception that complicates the integration of these procedures into broader public health frameworks (7).

From a public health perspective, one of the major epidemiological challenges is the limited visibility of aesthetic-surgery-related activity within routine administrative datasets.” While Germany also lacks comprehensive complication surveillance for many other surgical procedures, aesthetic surgery presents an additional challenge because a substantial proportion of procedures are performed in private outpatient and self-pay settings that are only incompletely represented in centralized reporting systems. Whereas international discourse often highlights the rise of minimally invasive, office-based techniques, the role of the inpatient sector remains epidemiologically opaque (8, 9). Secondary and tertiary hospitals remain the primary sites for surgical residency training and, crucially, serve as the “safety net” for managing complex surgical complications (10–12). Without accurate data on the volume and nature of hospital-based aesthetic surgery, public health officials cannot adequately map the burden of surgical complications or plan for workforce requirements (9).

The COVID-19 pandemic further complicated this landscape. The global suspension of elective surgeries during 2020–2021 created unprecedented backlogs across all surgical disciplines (13–15). However, preliminary data suggests that the aesthetic market may have exhibited a unique “rebound” effect, often colloquially termed the “Zoom Effect”, whereby remote work and increased digital self-exposure drove a surge in demand despite broader healthcare disruptions (16, 17). Understanding how elective surgical volumes fluctuated during and after the pandemic is essential for modeling healthcare system resilience and elective care recovery (15, 18).

To render these elective interventions visible within public health datasets, administrative frameworks such as the ICD-10 Z41.1 diagnosis code (“Procedures for purposes other than remedying health states”) were implemented. Theoretically, this code should allow health authorities to track procedures performed for non-medical reasons. However, the validity and utilization frequency of this diagnostic marker have never been scrutinized on a national scale (19, 20).

To address these gaps, we conducted the first nationwide longitudinal analysis of inpatient procedures commonly associated with aesthetic surgery in Germany over an 18-year period (2006–2023). By utilizing mandatory German Procedure Classification (OPS) codes, we tracked trends for four high-volume procedures: breast augmentation, liposuction, blepharoplasty, and abdominoplasty. These data were benchmarked against national estimates from the International Society of Aesthetic Plastic Surgery (ISAPS) to estimate the relative visibility of hospital-based procedures within the broader national aesthetic-surgery market and to assess whether observed patterns were consistent with apparent epidemiological migration from hospitals toward less visible care sectors (8, 21). This study specifically evaluates temporal patterns, COVID-19 impacts, and the performance of the Z41.1 code to determine if administrative hospital data can serve as a valid sentinel for the broader aesthetic market.

## Methods

### Study design and data source

This descriptive longitudinal epidemiologic analysis utilized nationwide administrative data (2006–2023) compiled by the Reimbursement Institute (Hürth, Germany). The dataset aggregates mandatory annual hospital quality reports by the Federal Joint Committee (Gemeinsamer Bundesausschuss, G-BA) pursuant to §136b SGB V. This provides broad longitudinal coverage of hospital-based inpatient procedures over an 18-year period, including statutory, privately insured, and self-paying inpatients treated within the German hospital sector.

### Case identification and coding strategy

Procedures were primarily identified using the German Procedure Classification (OPS) codes for four high-volume interventions: liposuction, breast augmentation, abdominoplasty, and blepharoplasty (Table 1).

**Table 1:** Procedure and diagnosis codes used in this study.

Procedure/Indication	Code
Procedures for purposes other than remedying health states	Z41.1 (ICD)
Abdominoplasty	5-911.0b (OPS)
Blepharoplasty	5-097 (OPS)
Breast augmentation	5-883 (OPS)
Liposuction	5-911.1 (OPS)

OPS (Operationen- und Prozedurenschlüssel) codes are used in the German healthcare system to classify surgical procedures for billing and statistical analysis. ICD-10 (International Classification of Diseases, 10th Revision) codes are used to document diagnoses. In this study, OPS codes were used to identify aesthetic procedures; ICD-10 Z41.1 was analyzed separately to examine national coding patterns but was not used for case selection.

Initially, the ICD-10 code Z41.1 was identified as a potentially superior administrative marker for isolating elective aesthetic cases from reconstructive ones. We hypothesized that this code would provide a clear diagnostic filter for non-medical interventions. Consequently, Z41.1 was analyzed in depth to evaluate its utility as a sentinel for the aesthetic market. However, due to observations of significant systemic underreporting and coding inconsistencies identified during the validation phase, OPS codes were maintained as the primary tool for case selection to ensure data sensitivity, while Z41.1 was analyzed separately to examine national coding patterns (19).

### Statistical analysis and benchmarking

Temporal trends were analyzed via univariate linear regression, with statistical significance defined at *p* < 0.05. Temporal trends were initially assessed using univariate linear regression. To further evaluate non-linear temporal dynamics and the potential impact of the COVID-19 pandemic, we performed exploratory segmented linear regression analyses for each procedure category and for total annual procedural volume. Calendar year was modeled as a continuous variable, with 2020 specified as the interruption point. The models estimated the pre-2020 annual slope, the immediate 2020 level change, and the post-2020 slope change. Given the limited number of available reporting years, these analyses were interpreted as exploratory assessments of structural change rather than causal interrupted time-series models. Heteroscedasticity-robust confidence intervals were calculated. Regional heterogeneity was assessed descriptively using geospatial mapping and provider-volume stratification (stratified into quartiles of high-, medium-, and low-volume Quantitative analyses were conducted using SPSS Statistics version 30.0 (IBM Corp., Armonk, NY). To quantify the “hospital share” of the total market, inpatient volumes were benchmarked against national estimates provided by the International Society of Aesthetic Plastic Surgery (ISAPS) Global Survey Reports for the years 2010, 2016, and 2023 (21). Because ISAPS data are based on voluntary survey reporting and national extrapolation, they were not treated as a gold-standard denominator. Rather, ISAPS estimates were used as contextual external benchmarks to approximate the relative visibility of hospital-based procedures within the broader national aesthetic surgery market. Accordingly, calculated hospital-share percentages were interpreted as directional indicators of surveillance coverage rather than precise capture rates. Geospatial distributions and regional volume variations were visualized using Datawrapper (Berlin, Germany).

### Ethical considerations

All data were processed in an anonymized and aggregated format in accordance with national data protection standards. No institutional or patient-identifiable information was accessible. Pursuant to the Declaration of Helsinki and the secondary nature of the administrative data, formal ethical approval was not required.

## Results

From 2006 to 2023, German hospitals recorded 352,468 procedures across the four study categories: blepharoplasty (*n* = 167,756), liposuction (*n* = 92,929), abdominoplasty (*n* = 57,759), and breast augmentation (*n* = 34,024). During this period, concurrent diagnostic coding with ICD-10 Z41.1 occurred in 0.4% of cases (*n* = 1,537). Z41.1 usage rose from 78 cases in 2006 to 119 in 2023, with linear regression showing no significant temporal trend (*p* = 0.90).

**Figure 1:**
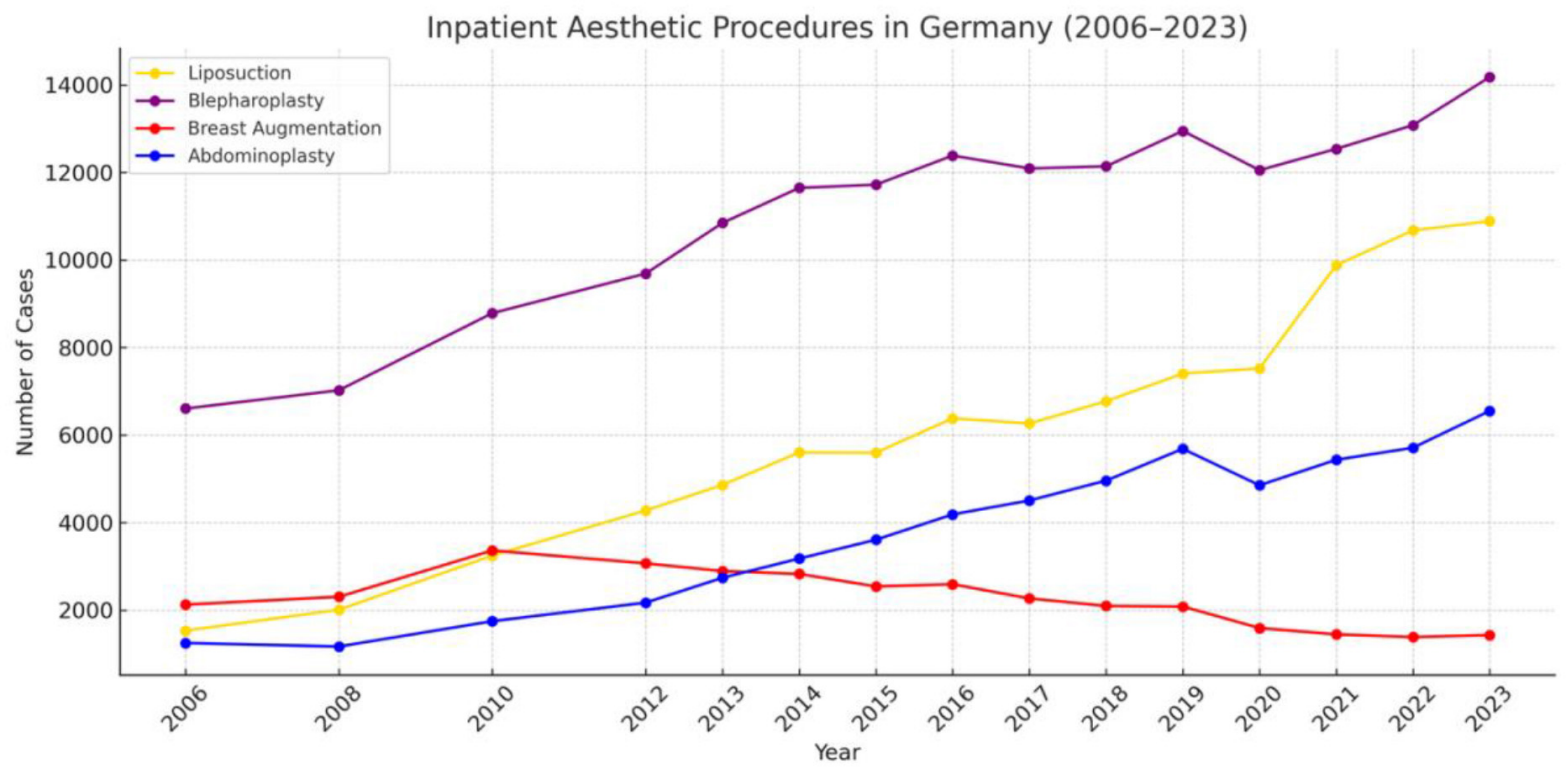
Inpatient aesthetic-surgery procedures in Germany, 2006–2023. The chart displays annual case volumes for liposuction, blepharoplasty, breast augmentation, and abdominoplasty.

Annual inpatient volume increased from 11,593 procedures in 2006 to 33,170 in 2023. In 2006, the distribution was: blepharoplasty (*n* =6,606), breast augmentation (*n* = 2,127), liposuction (*n* = 1,532), and abdominoplasty (*n* = 1,250). By 2023, annual volumes were: blepharoplasty (*n* = 14,181), liposuction (*n* = 10,888), abdominoplasty (*n* = 6,552), and breast augmentation (*n* = 1,430). Temporal dynamics for each procedure, including the impact of the COVID-19 pandemic, are illustrated in Figure 1.

### Diagnostic coding with ICD-10 Z41.1

Cases coded under ICD-10 Z41.1 rose from 78 in 2006 to 119 in 2023, with a peak of 320 cases in 2013. Univariate linear regression showed no significant temporal trend (slope = −0.45 cases/year; 95% CI−7.97 to +7.08; *p* = 0.90; R^2^ = 0.001) (See Supplementary 1).

### Abdominoplasty

**Figure 2:**
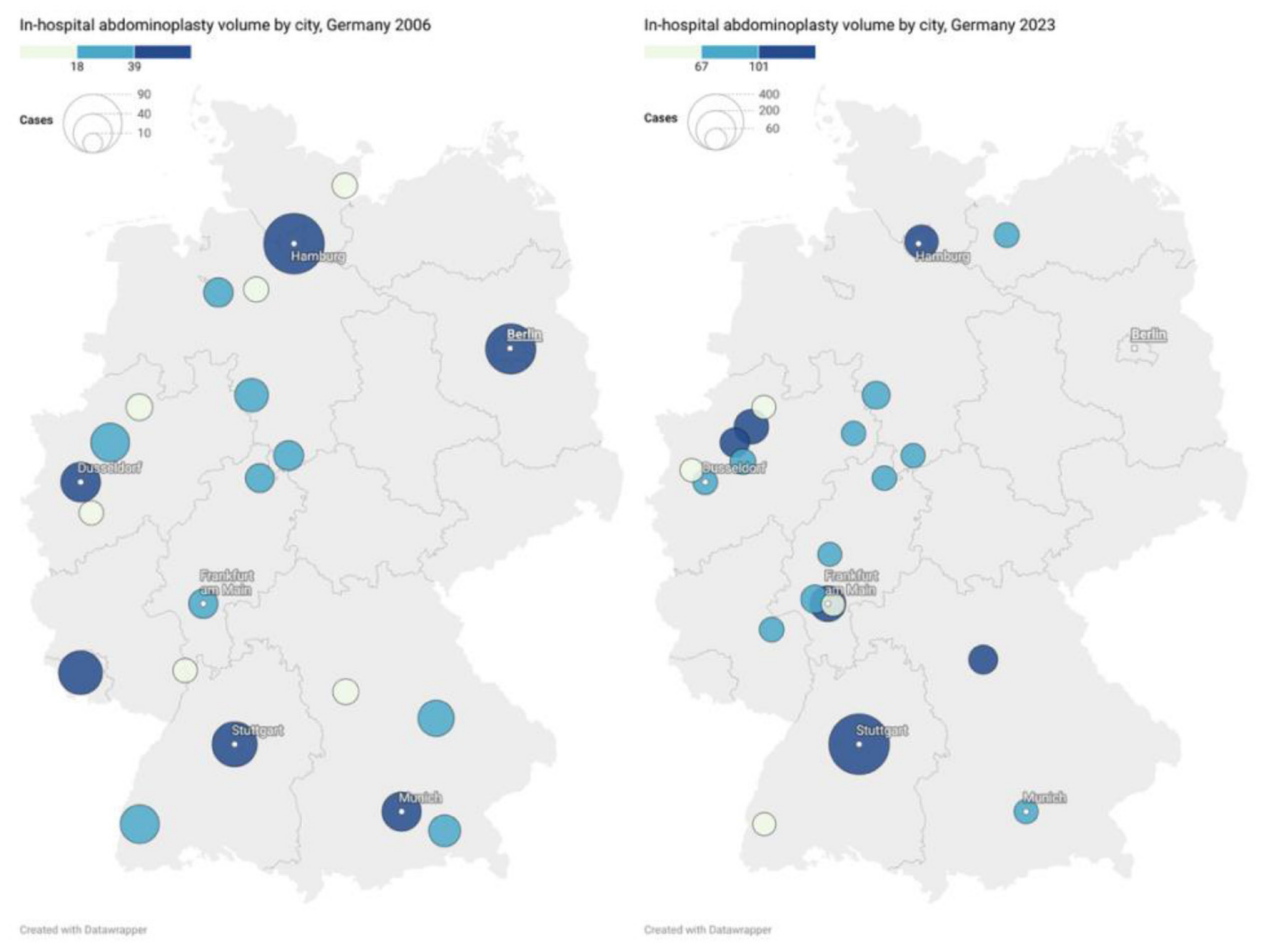
In-hospital abdominoplasty volume by city, Germany, 2006 vs. 2023. This figure displays the geographic distribution and volume of inpatient abdominoplasties performed across German cities in 2006 **(left)** and 2023 **(right)**. Circle size reflects absolute case counts, while color intensity indicates procedural volume, with darker blue signifying higher activity. Between 2006 and 2023, procedure counts increased substantially and concentrated in a smaller number of urban centers. Düsseldorf and Stuttgart emerged as major hubs for hospital-based abdominoplasty, while several previously active locations showed relative contraction.

Inpatient abdominoplasty volumes increased from 1,250 in 2006 to 6,552 in 2023 (+424%). Linear regression confirmed a significant upward trend (*p* < 0.001; slope = 332; 95% CI +290 to +374; R^2^ = 0.96). Despite a transient decline in 2020 (*n* = 4,494), volumes increased annually thereafter. Regionally, Hamburg recorded the highest volume in 2006 (*n* = 96), while Stuttgart recorded the highest volume in 2023 (*n* = 472). By 2023, annual totals also exceeded 100 cases in centers including Frankfurt, Recklinghausen and Erlangen (Figure 2).

### Blepharoplasty

Inpatient blepharoplasty procedures increased from 6,606 in 2006 to 14,181 in 2023 (+115%). Linear regression confirmed a significant upward trend (*p* < 0.001; slope = 407; 95% CI +322 to +492; R^2^ = 0.89), with a transient decline in 2020. Geographically, Munich recorded the highest case volumes at both time points. By 2023, volumes in Frankfurt and Stuttgart increased, while totals in previously high-volume centers, such as Trier and Hagen, declined.

### Breast augmentation

**Figure 3:**
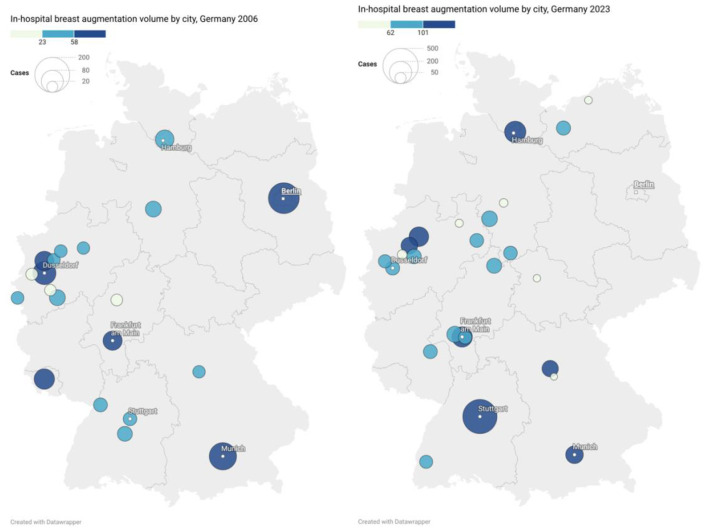
In-hospital breast augmentation volume by city, Germany, 2006 vs. 2023. This figure illustrates the distribution and procedural volume of inpatient breast augmentations across German cities in 2006 **(left)** and 2023 **(right)**. Circle size corresponds to absolute case counts, while color reflects procedural intensity, with darker blue indicating higher volumes. Compared to 2006, the 2023 map reveals an overall reduction in volume and a marked shift toward fewer, more centralized providers. Major urban centers such as Munich, Stuttgart, and Düsseldorf maintained higher volumes, while formerly active regions experienced contraction.

Inpatient breast augmentation volumes decreased from 2,127 in 2006 to 1,430 in 2023 (−32.8%). This downward trend was statistically significant (*p* = 0.004; slope = −83.8; 95% CI−136 to−31; R^2^ = 0.48). Peak activity occurred between 2010 and 2012, with a sustained decline following 2019. In 2006, leading centers included Berlin, Munich, and Düsseldorf; by 2023, Stuttgart recorded the highest volume (*n* = 472 across three sites), while volumes in several formerly prominent centers declined (Figure 3).

### Liposuction

**Figure 4:**
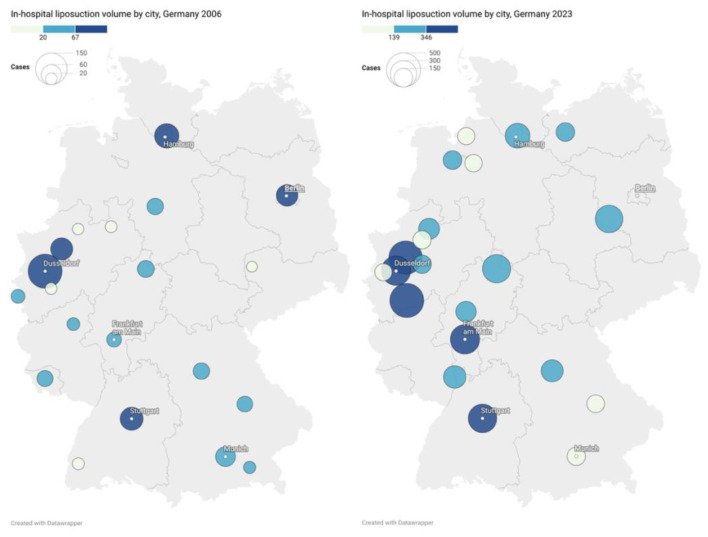
In-hospital liposuction volume by city, Germany, 2006 vs. 2023. These maps illustrate the geographic distribution and procedural volume of inpatient liposuctions performed in German hospitals in 2006 **(left)** and 2023 **(right)**. Each circle represents a city with at least one reporting hospital; the size of the circle corresponds to the absolute number of cases, while color reflects procedural intensity, with darker blue indicating higher volumes. Over the 18-year period, liposuction volumes increased substantially and became more centralized, particularly in regions such as Düsseldorf, Frankfurt am Main, and Stuttgart.

Inpatient liposuction volumes rose from 1,532 cases in 2006 to 10,888 in 2023 (+611%). Regression analysis confirmed a significant upward trend (*p* < 0.001), averaging 543 additional cases annually (95% CI +473 to +613; R^2^ = 0.96). Volumes plateaued in 2020 (*n* = 7,654) before increasing from 2021 (*n* = 9,331) through 2023. Regionally, the highest volumes shifted from Düsseldorf in 2006 (*n* = 173) to Bonn (*n* = 497) and Essen (*n* = 478) in the year 2023 (Figure 4).

### International benchmarking with ISAPS data

**Figure 5:**
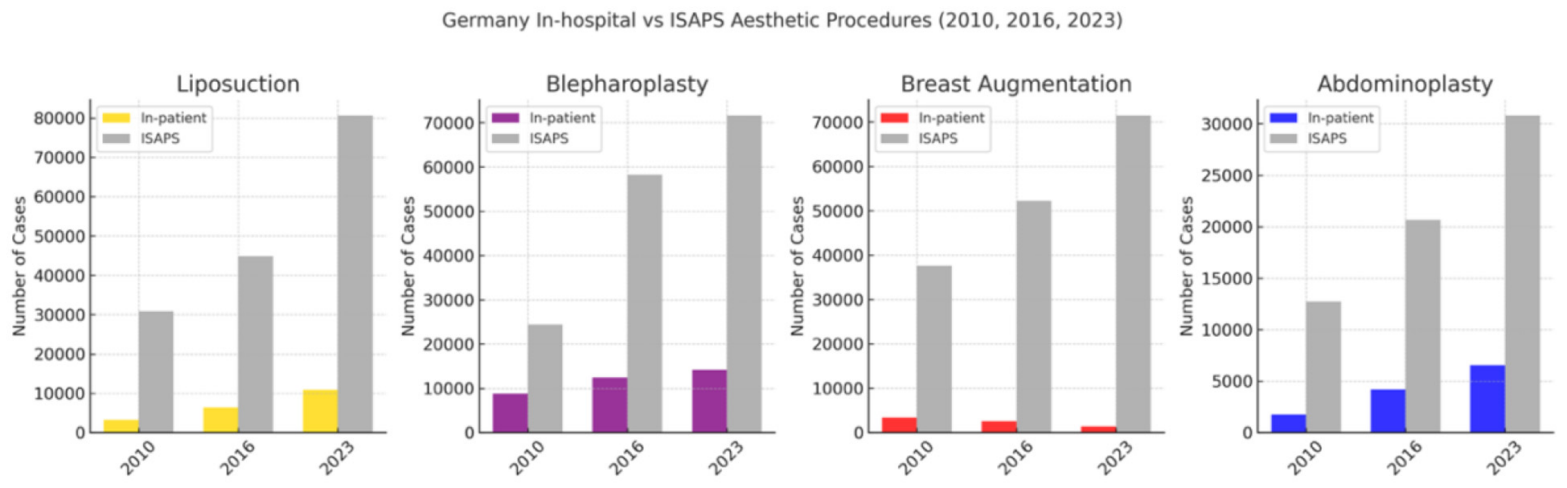
The figure compares German in-hospital case volumes (colored bars) with ISAPS-reported totals for 2010, 2016, and 2023 (gray bars) for each of the four leading aesthetic procedures.

Comparison between hospital data and ISAPS national estimates (2010–2023) suggested divergent patterns of relative hospital-sector visibility across procedures. For liposuction, inpatient share of national volume increased from 10.5% (*n* = 3,247) to 13.5% (*n* = 10,888). Similarly, abdominoplasty hospital share rose from 13.7% (*n* = 1,748) to 21.3% (*n* = 6,552) (Figure 5).

Conversely, blepharoplasty share declined from 36.0% (*n* = 8,786) to 19.8% (*n* = 14,181). The most pronounced change was observed for breast augmentation, where the hospital-to-ISAPS ratio decreased from 8.9% (*n* = 3,364) in 2010 to 2.0% (*n* = 1,430) in 2023. These shifts in institutional share were non-linear, with intermediate 2016 shares recorded at 14.2% for liposuction, 21.3% for blepharoplasty, 20.3% for abdominoplasty, and 5.0% for breast augmentation (Table 2).

**Table 2:** Hospital figures reflect inpatient procedures coded in German reimbursement data; ISAPS figures represent total national estimates for all surgical settings.

Year	Source	Liposuction	Blepharoplasty	Breast augmentation	Abdominoplasty
**2010**	Hospital	3 247	8 786	3 364	1 748
	ISAPS	30 861	24 397	37 653	12 738
	Hospital share (%)	10.5 %	36.0 %	8.9 %	13.7 %
**2016**	Hospital	6 384	12 387	2 593	4 188
	ISAPS	44 836	58 245	52 209	20 678
	Hospital share (%)	14.2 %	21.3 %	5.0 %	20.3 %
**2023**	Hospital	10 888	14 181	1 430	6 552
	ISAPS	80 591	71 604	71 464	30 789
	Hospital share (%)	13.5 %	19.8 %	2.0 %	21.3 %

Hospital share indicates the proportion of the ISAPS total captured by inpatient data.

Exploratory segmented regression with 2020 specified as the interruption point demonstrated distinct procedure-specific temporal patterns (Supplementary Table 2). Total procedural volume increased significantly before 2020 by 1,309.6 cases per year (95% CI 1,145.9 to 1,473.3; *p* < 0.001), followed by a significant immediate level decrease in 2020 (-3,361.2 cases; 95% CI−4,920.7 to−1,801.7; *p* < 0.001) and a steeper post-2020 annual increase of 2,263.3 cases per year (95% CI 1,901.0 to 2,625.6; *p* < 0.001). Abdominoplasty and blepharoplasty both showed significant pre-2020 growth and significant immediate 2020 declines, followed by renewed post-2020 increases. Liposuction differed from this pattern: although pre-2020 volumes increased significantly by 463.2 cases per year (95% CI 422.7 to 503.7; *p* < 0.001), there was no significant immediate 2020 level decrease (*p* = 0.782), and the post-2020 slope increased to 1,088.9 cases per year (95% CI 466.7 to 1,711.1; *p* = 0.003). Breast augmentation showed a significant immediate 2020 decline (-839.7 cases; 95% CI−1,362.0 to−317.4; *p* = 0.005), with continued post-2020 decrease.

## Discussion

Germany's experience illustrates the challenges faced by health systems with centralized hospital reporting but fragmented outpatient surveillance. Inpatient data, while unrepresentative of absolute national volumes, remain a vital sentinel for institutional resource allocation. In the context of Germany's Leistungsgruppen framework, the 611% rise in inpatient liposuction suggests a growing overlap between hospital-based procedural activity and procedures commonly associated with elective aesthetic care. Integrating private-sector activity into national frameworks is essential to monitor the equitable distribution of surgical resources.

Benchmarking against ISAPS estimates suggests limited hospital-sector visibility for selected procedures: in 2023, inpatient hospital volumes corresponded to 2.0% of estimated national breast augmentation volume and 13.5% of estimated national liposuction volume. Because outpatient and office-based activity was not directly measured, these figures should be interpreted as hospital-to-ISAPS ratios rather than precise capture rates. Nevertheless, the magnitude and procedure-specific pattern of these discrepancies are consistent with substantial undercapture of aesthetic-surgery-related activity by mandatory hospital reporting. A primary methodological takeaway is the failure of the ICD-10 code Z41.1 for surveillance. Despite its theoretical role in identifying procedures performed for purposes other than remedying health states, Z41.1 was absent in more than 99% of procedure-coded cases. Because the German DRG system prioritizes procedural (OPS) codes for reimbursement, Z41.1 lacks utility for case identification (19). ICD-10-GM codes, by contrast, document diagnoses and are relevant to reimbursement only when they contribute to case grouping, comorbidity adjustment, or medical necessity. Z41.1 occupies an ambiguous position within this framework: although conceptually suited to identify procedures performed for purposes other than remedying health states, it does not define the intervention itself, and it appears to carry limited practical coding incentive in routine hospital documentation. Its use in only 0.4% of procedure-coded cases, without a meaningful temporal trend, therefore, suggests not merely underreporting but a structural mismatch between the intended epidemiologic function of the code and the operational logic of administrative hospital coding. This serves as a warning of extreme selection bias for researchers; until the full implementation of ICD-11, procedural codes remain the most sensitive available mapping tool in this dataset, although they cannot distinguish aesthetic from reconstructive intent (20, 22).

This limitation is not unique to Germany. In the United States, inpatient procedures are classified through ICD-10-PCS, while CPT and HCPCS codes are central to physician/service and item-based billing. In England, OPCS-4 is used to classify interventions and surgical procedures, whereas ICD-10 is used for diseases and other health conditions. Across these systems, procedure codes generally describe what was done more reliably than why it was done, underscoring the need for linked diagnosis, indication, payer, and outcome data when distinguishing aesthetic from reconstructive surgery.

Importantly, this limitation is not unique to aesthetic surgery. Germany currently lacks comprehensive complication surveillance systems for many surgical procedures. However, aesthetic surgery differs in that a considerable proportion of procedural activity occurs outside statutory reimbursement pathways and routine hospital-centered reporting structures, thereby further limiting denominator visibility. From an infection-control and patient-safety perspective, the implications of this surveillance gap are primarily epidemiological. The present study does not measure surgical site infections, complication rates, or cluster outbreaks directly. However, valid estimation of any complication rate requires both a numerator, such as infections, readmissions, or reoperations, and a reliable denominator representing the population or procedures at risk. The inability to consistently identify these procedures in administrative data as a denominator may allow complication clusters to remain underrecognized until they present in tertiary care settings.

The geographic analysis should be interpreted as a descriptive mapping of hospital-reported procedural activity rather than as a causal analysis of regional demand. Nevertheless, several contextual factors may plausibly explain the observed concentration of procedures in selected urban centers. First, large cities and metropolitan regions have greater population catchments, transport accessibility, and patient inflow from surrounding regions, which may increase the visibility of elective and aesthetic-surgery-related procedures in hospital reporting. Second, regional clustering may reflect the presence of specialized private clinics, Belegarzt departments, or §108/109 SGB V institutions that operate within the hospital reporting framework and are therefore captured in the present dataset despite serving a partly private or self-pay market. Third, university hospitals and tertiary referral centers may contribute to regional visibility through reconstructive case mix, complication management, or procedure-specific expertise, whereas purely office-based private practice remains largely invisible in these data. Finally, procedure-specific factors may differ across regions: for example, inpatient liposuction volumes may partly reflect regional referral structures for lipedema care rather than purely aesthetic demand. These contextual variables could not be formally modeled because the dataset lacks patient residence, catchment denominators, provider ownership structure, payer status, outpatient-sector volumes, socioeconomic indicators, and indication-level case mix. Another factor to keep in mind is broader workforce dynamics: the number of board-certified plastic surgeons doubled between 2010 and 2022, outstripping the growth of the general physician workforce (23, 24).

### Pandemic disruption and Post-COVID resilience

The COVID-19 pandemic coincided with a transient decline in several inpatient procedure categories in 2020, consistent with broader reports of reduced elective surgical activity during the early pandemic period (13–15). In contrast to abdominoplasty and blepharoplasty, liposuction showed no significant immediate 2020 level decline in segmented regression and demonstrated a steeper post-2020 slope, suggesting that the pandemic did not interrupt the inpatient liposuction trajectory in the same way as other procedure categories. This pattern is compatible with international reports of rapid recovery in surgical volumes by 2021 following initial lockdowns and recommendations to postpone non-urgent care (14–16).

By 2021, inpatient volumes for liposuction, blepharoplasty, and abdominoplasty exceeded their 2019 levels. This may reflect recovery of institutional throughput, changes in patient demand, shifts in case selection, or changes in the composition of procedures remaining within the hospital sector. Conversely, breast augmentation continued its pre-existing downward trajectory after 2020. Explanations such as procedural invasiveness, evolving patient priorities during remote work, or the so-called “Zoom Effect” remain plausible but cannot be directly tested in the present dataset (17). Therefore, these mechanisms should be interpreted as hypotheses supported by prior literature rather than as causal conclusions from the administrative data.

### Comparison with prior national and international data

Our findings are consistent with substantial undercapture of the broader national aesthetic-surgery market by inpatient administrative data. This pattern was most pronounced for breast augmentation, for which inpatient hospital volume corresponded to only 2.0% of the ISAPS national estimate in 2023. However, because the present dataset does not measure private outpatient or office-based procedures directly, this finding should be interpreted as indirect evidence of reduced hospital-sector visibility rather than definitive proof of sectoral e. The observed discrepancies are consistent with literature describing increasing delivery of aesthetic procedures in ambulatory and office-based settings (3, 9). Consequently, traditional hospital-based datasets appear insufficient as standalone proxies for national procedural trends. Benchmarking suggests that hospital visibility varies substantially by procedure: while breast augmentation and blepharoplasty appear less visible within inpatient reporting, hospital-based liposuction and abdominoplasty retain a larger and increasing institutional footprint. In contrast to the coding limitations identified in our dataset, the US. Tracking Operations and Outcomes for Plastic Surgeons (TOPS) database provides a benchmark for mandatory, outcomes-based reporting. Analyzing over 214,000 patients between 2008 and 2019, TOPS data demonstrated a low overall incidence of perioperative complications, including deep surgical-site infections (0.2%) and venous thromboembolism (0.1%) (10). However, the TOPS registry highlights specific vulnerabilities that align with our inpatient observations: patients undergoing abdominoplasty more frequently required emergency room visits or unplanned readmissions compared to other aesthetic procedures. Furthermore, the TOPS data confirms that pursuit of combined aesthetic procedures is significantly associated with increased risk for unplanned healthcare utilization.

While registries like TOPS provide vital insights into underreported outcomes and the impact of comorbidities (e.g., diabetes and BMI), the lack of similar, mandatory, cross-sectoral reporting in Europe remains a barrier to accurate epidemiological mapping and infection surveillance (25). Bridging this gap is essential to replicate the safety-benchmarking capabilities seen in the North American model.

### Combined aesthetic procedures and the downstream role of tertiary care

The rise of combined aesthetic procedures, such as “Mommy Makeovers,” reflects an evolving practice pattern in which multiple interventions are performed during a single operative episode, often with the aim of consolidating recovery time (26, 27). From a surveillance perspective, however, this creates an important methodological challenge. Administrative data are coded by procedure rather than by unique patient encounter, and the present dataset cannot determine whether liposuction, abdominoplasty, breast surgery, or other procedures were performed as isolated interventions or as part of combined operations. This distinction is clinically relevant because combined procedures may be associated with different operative durations, complication profiles, and postoperative resource requirements than single procedures (26–28). In US. registry data, liposuction frequently functions as an adjunct procedure, and combined aesthetic procedures have been associated with increased unplanned healthcare utilization (10). Procedure-code counts should therefore be interpreted cautiously, as they may overrepresent the number of coded procedures while underrepresenting the complexity of individual operative episodes.

At the same time, a growing share of primary aesthetic activity appears to occur outside traditional tertiary hospital environments, including ambulatory, office-based, private-sector, and international settings (3, 9, 11). This shift may reduce hospital-based exposure to primary elective aesthetic procedures, yet it does not eliminate the need for hospital-based management when complications occur. Serious complications after liposuction and body-contouring procedures remain well documented, including in German experience, and large-volume or complex body-contouring procedures may require escalation to hospital-based care when complications occur (29, 30). Together with evidence that complications after outpatient cosmetic surgery can generate substantial hospital charges, with a US study reporting median hospital admission charges of $35,637 for acute postoperative infections and hemorrhages, these data support the plausibility of a downstream safety-net function for tertiary plastic surgery services (31).

Several studies from other healthcare systems directly support this concern. In Switzerland, Klein et al. reported 109 patients presenting with complications after cosmetic surgery tourism; infection was the most common complication, followed by wound breakdown and pain or discomfort, and 34.8% of patients required inpatient treatment with a mean hospital stay of 5.2 days (32). In a later series, Hummel et al. identified 228 patients treated at University Hospital Zurich for complications after aesthetic procedures performed abroad or in Switzerland. Most complications followed procedures performed abroad, but 16% occurred after procedures performed domestically in Switzerland; breast surgery was the most common primary procedure, followed by body contouring and facial surgery, and total treatment costs over the study period reached $795,574 (33).

Similar patterns have been reported elsewhere. Belza et al. examined 40 patients managed at a single academic tertiary referral center over 5 years for complications after cash-paid aesthetic procedures performed at outside facilities (34). In Bahrain, Farhan et al. assessed the economic impact of cosmetic surgery tourism on a tertiary center and reported that 30 patients presented with complications, most commonly after abdominoplasty; surgical-site infection was the most frequent complication (35).

Data from the United Kingdom further illustrate the public-system burden. Magness et al. described patients referred to an NHS plastic surgery service with serious complications of private-sector cosmetic surgery during the second COVID-19 wave, when public-sector operative capacity was largely restricted to emergency and oncologic care. Presenting complications included infection and wound dehiscence, two patients presented in septic shock, one developed necrotizing panniculitis, and one patient died perioperatively. The median number of NHS surgical procedures was 2.5, and all survivors had unsatisfactory aesthetic outcomes (36). Henry et al. reported an average National Health Service cost of £5,882.54 per patient after cosmetic surgery tourism complications (37). Roberts et al. reported 81 patients presenting to NHS Scotland with complications after cosmetic surgery tourism over a five-year period, with a total cost of £755,559.68 and an average cost of £9,327.90 per patient (38). Whiteman et al. (39) similarly described a rising NHS burden from postoperative complications after cosmetic surgery performed abroad and non-surgical procedures performed in the United Kingdom. Campbell et al. (40) provide complementary evidence from the international medical-tourism literature, showing the scale and complexity of cross-border aesthetic surgery pathways.

### Public health implications: workforce and training

These studies support the broader public-health concern that aesthetic procedures performed outside mandatory hospital reporting frameworks may still generate downstream hospital workload, including emergency assessment, inpatient care, revision surgery, infection management, implant removal, wound care, and complex reconstructive salvage. Importantly, these interventions may not be sufficient to restore what the patient originally sought from the index procedure: a satisfactory aesthetic outcome. However, this safety-net mechanism cannot be directly tested in the present dataset. We cannot determine what proportion of inpatient cases represented primary aesthetic-surgery-related procedures, planned revisions, staged operations, or secondary management of complications after surgery performed elsewhere. The observed discrepancy between mandatory hospital reporting and broader national estimates should therefore be interpreted as evidence of limited surveillance visibility rather than as a direct measure of complication burden.

This has implications for workforce planning and surgical education. If primary aesthetic procedures increasingly occur in private, office-based, ambulatory, or international settings, while complications return to tertiary or public hospital services, academic departments may face a growing mismatch between procedural exposure and downstream responsibility. Residents may encounter fewer planned primary aesthetic procedures and proportionally more unplanned revision, infection management, implant removal, wound care, and reconstructive salvage. Although complication management is educationally valuable, it cannot replace structured exposure to indication setting, patient selection, aesthetic planning, operative execution, and longitudinal follow-up of primary aesthetic procedures. The same issue may affect consultants and institutional expertise: lower volumes of primary aesthetic procedures in hospital-based environments may reduce opportunities to maintain procedure-specific experience, supervise trainees, and develop standardized perioperative pathways. While the present study cannot test a volume–outcome relationship in aesthetic surgery, broader surgical literature suggests that higher hospital and surgeon volume are often associated with improved outcomes, while acknowledging procedure-specific variation and methodological limitations (41–43).

This creates a system-level mismatch: patients may experience both medical complications and unsatisfactory aesthetic outcomes; residents and consultants may see fewer planned primary procedures but more salvage surgery; and public or tertiary hospitals may absorb downstream complication care without having been involved in the original indication setting, patient selection, operative planning, or follow-up pathway. Maintaining adequate domestic training pathways is therefore relevant for workforce planning, quality assurance, and patient safety. If confirmed in linked cross-sectoral datasets, the apparent redistribution of aesthetic procedures across care settings would support regional networks connecting academic departments with high-volume ambulatory and private-sector providers. Such models could preserve exposure to primary aesthetic procedures while ensuring transparent complication reporting, shared quality standards, and structured referral pathways. International bodies have emphasized the importance of aesthetic surgery training during plastic surgery residency (12, 44, 45), and resident-led aesthetic clinics suggest that structured academic exposure can be delivered safely when appropriate supervision and governance are in place (46, 47). Future research should link hospital procedural data with claims data, emergency presentations, complication diagnoses, payer information, operative-setting identifiers, and prospective aesthetic surgery registries to quantify downstream complication burden, costs, patient outcomes, and effects on training exposure.

### Limitations

This study is subject to several structural and methodological limitations inherent in the use of secondary administrative data. Most significantly, the current German hospital reporting system does not allow for a direct linkage between diagnostic (ICD-10) and procedural codes at the patient level. This prevents a definitive distinction between aesthetic and reconstructive indications for procedures with dual utility. Consequently, while we can track procedural volumes, we cannot definitively quantify the underlying clinical intent or the proportion of cases that were strictly elective. We initially considered ICD-10 Z41.1, “procedures for purposes other than remedying health states,” as a potential proxy marker for explicitly aesthetic indications and as a possible basis for sensitivity analyses. However, Z41.1 was coded in only 0.4% of all included cases and showed no meaningful temporal pattern, indicating that it reflects inconsistent local coding behavior rather than a reliable indication-specific subset. Therefore, Z41.1 cannot be interpreted even as a conservative lower-bound estimate of aesthetic surgery volume. In the absence of patient-level diagnostic linkage, payer information, clinical indication fields, or longitudinal claims data, no valid sensitivity analysis can separate aesthetic from reconstructive cases within the present dataset. Such analyses would require either a dataset that directly links procedures to diagnoses and indications or linkage of the present hospital quality-report data with complementary claims-based or registry datasets.

The risk of indication misclassification likely differs by procedure. Abdominoplasty spans both cosmetic and reconstructive indications, including purely aesthetic body contouring, post-bariatric reconstruction, symptomatic pannus, and abdominal-wall-related indications. Blepharoplasty carries a similarly substantial risk of indication ambiguity, as it may be performed for reimbursable functional indications under predefined criteria of visual impairment, but may also be undertaken for aesthetic reasons, often in outpatient or day-surgery settings under local anesthesia. For breast augmentation, the risk of inadvertently capturing post-mastectomy or standard reconstructive implant-based breast reconstruction is comparatively limited, because these procedures are generally represented by distinct breast reconstruction and implant-exchange OPS codes rather than the primary augmentation code used in this analysis. Nevertheless, some medically indicated implant procedures, for example in congenital asymmetry or deformity correction, cannot be fully excluded. Liposuction is particularly vulnerable to indication ambiguity, especially given the increasing clinical and public attention to lipedema in Germany; rising inpatient liposuction volumes may therefore reflect medically indicated treatment of lipedema rather than growth in purely aesthetic body-contouring surgery. This is even further complicated by the fact that public health care insurance only covers liposuctions as a treatment option for lipedema in certified centers, among which to date to our knowledge there are no office-based practices which essentially means that almost all liposuctions paid for by insurance will be included in this dataset.

These procedure-specific differences introduce potential classification bias. Reconstructive or medically indicated cases may lead to overestimation of aesthetic inpatient activity, whereas the non-capture of private outpatient procedures billed outside the hospital reporting framework likely leads to substantial underestimation of the total national aesthetic surgery market.

The benchmarking analysis is also limited by the structure of the ISAPS Global Survey data. Although ISAPS provides one of the few internationally comparable sources for aesthetic procedure volumes, these estimates are derived from voluntary survey-based reporting and are therefore subject to uncertainty regarding completeness and representativeness. Potential response biases include selective participation by specific provider groups, overrepresentation of high-volume or society-affiliated surgeons, underrepresentation of smaller private practices, and variation in national response rates across survey years. In addition, changes in respondent composition, extrapolation assumptions, or procedure definitions may affect temporal comparability. Therefore, the calculated hospital-share estimates should not be interpreted as precise capture rates or definitive denominators for the German aesthetic surgery market. Instead, they provide a contextual benchmark indicating the approximate scale of the discrepancy between mandatory hospital reporting and the broader, largely private aesthetic surgery sector. While this uncertainty limits confidence in the exact percentage estimates, it does not negate the central finding that hospital administrative data represent only a sentinel fraction of total national procedural activity.

Thirdly, the suppression of small case numbers (1–3 cases per hospital) in accordance with G-BA data protection regulations introduces a systematic underestimation bias. This effect is particularly relevant for low-volume providers and potentially lead to an underrepresentation of decentralized care structures.

Although additional segmented time-series analyses were performed to explore non-linear trends and potential structural breaks around the COVID-19 pandemic, the statistical modeling remains constrained by the structure of the available data. The annual time series comprised only 18 observations per procedure, limiting the robustness of highly parameterized time-series models. Similarly, although regional heterogeneity was visualized and described, formal multilevel modeling was not feasible without stable patient-level or provider-level covariates, catchment denominators, outpatient-sector volumes, payer information, and indication-specific case-mix. Mixed-effects models in this context would risk overfitting and could imply causal or explanatory precision. Therefore, the maps should be interpreted as visualizations of where procedures were reported, not as population-adjusted regional incidence maps or as evidence of regional differences in underlying patient demand.

Furthermore, our dataset is restricted to inpatient care and ambulatory surgery performed specifically within the hospital setting. Because a substantial and growing share of German aesthetic practice occurs in private, office-based clinics billed under the *Gebührenordnung für Ärzte* (GOÄ), these volumes are not captured in the national Quality Reports. There is currently no centralized registry for these private-sector interventions, meaning our data likely represent a sentinel share of the total national volume rather than a comprehensive demographic map. Moreover, this dataset does not track outpatient procedures. Other datasets, such as those provided by the Research Data Center for Health (FDZ Gesundheit), include both inpatient and outpatient claims for patients covered by statutory health insurance and allow patient-level linkage between diagnoses, procedures, and outcomes. In contrast to the aggregated hospital quality reports used in the present study, FDZ datasets may also permit identification of concurrent procedures performed during the same encounter, thereby enabling future analyses of combined aesthetic procedures, longitudinal complication rates, infection risks, and downstream healthcare utilization.

The administrative nature of the hospital reports also restricts clinical granularity. As these data are compiled for regulatory rather than research purposes, patient-level details, including age, specific comorbidities, and longitudinal outcomes, are unavailable. The reporting of procedural codes rather than unique patient encounters further precludes the evaluation of staged procedures or revision rates. While age-standardized rates using the WHO standard population would improve international comparability, the lack of age-specific granularity in the raw German data remains a hurdle.

Finally, diagnostic coding remains a major challenge for surgical surveillance. In many cases, specific conditions like breast implant–associated anaplastic large cell lymphoma (BIA-ALCL) were not uniquely identifiable in the earlier years of our study, as they were often grouped under broader systemic ALCL codes. Although some improvements have been implemented, the transition to ICD-11 represents a necessary shift toward the standardized, granular coding required for robust patient safety monitoring and international workforce planning. The transition to ICD-11 offers significant improvements over ICD-10 for the surveillance of aesthetic procedures. ICD-11 introduces greater diagnostic granularity and allows for the use of extension codes that can more precisely define the context and indication of procedures, including distinctions between aesthetic and medically indicated interventions. Furthermore, ICD-11 enhances the ability to link procedures with complications and outcomes, thereby improving the capacity for longitudinal patient safety monitoring (20).

## Conclusion

This 18-year analysis shows that mandatory German hospital data provide only a limited and procedure-specific view of procedures commonly associated with aesthetic surgery. Inpatient breast augmentation represented only a small proportion of national ISAPS estimates by 2023, whereas hospital-based liposuction and abdominoplasty increased substantially over the study period. These findings should be interpreted as evidence of procedure-specific hospital-sector visibility rather than as a comprehensive measure of the national aesthetic-surgery market or direct proof of outpatient migration. Nevertheless, the marked discrepancy between mandatory hospital reporting and external national estimates is consistent with substantial undercapture of aesthetic-surgery-related activity by routine administrative datasets.

The very low use of ICD-10 Z41.1 highlights a major limitation of current diagnostic coding for indication-specific surveillance. Administrative procedure codes identify what was performed, but not reliably why it was performed. As a result, current hospital datasets cannot adequately distinguish aesthetic from reconstructive intent, identify combined procedures at the patient level, or link procedures to complications across care sectors. This limitation has important theoretical implications for infection surveillance, complication monitoring, workforce planning, and training because reliable rate estimation requires a valid procedural denominator. However, the magnitude of downstream complication burden, safety-net activity, and training effects cannot be quantified from the present dataset alone.

More robust surveillance will require integrated cross-sectoral registries, improved indication coding, and linkage between procedures, diagnoses, payer sector, operative setting, complications, and outcomes. Such infrastructure would allow health systems to better quantify elective aesthetic surgery, distinguish primary procedures from revision or complication management, monitor patient-safety events, and assess how private-sector, office-based, international, and hospital-based procedural activity interact. Until such linkage is available, German hospital data should be regarded as a sentinel source for selected institutional trends rather than a complete epidemiological map of the aesthetic surgery market.

## Data Availability

The original contributions presented in the study are included in the article/Supplementary material, further inquiries can be directed to the corresponding author.
