# Supplementary figures and images for: Gaps in public health surveillance of elective surgery: a national analysis of inpatient data, COVID-19 impact, and coding validity in Germany (2006–2023)
Source: Front Public Health. 2026 Jun 17;14:1813408. doi: 10.3389/fpubh.2026.1813408 (PMC13319030; doi:10.3389/fpubh.2026.1813408)

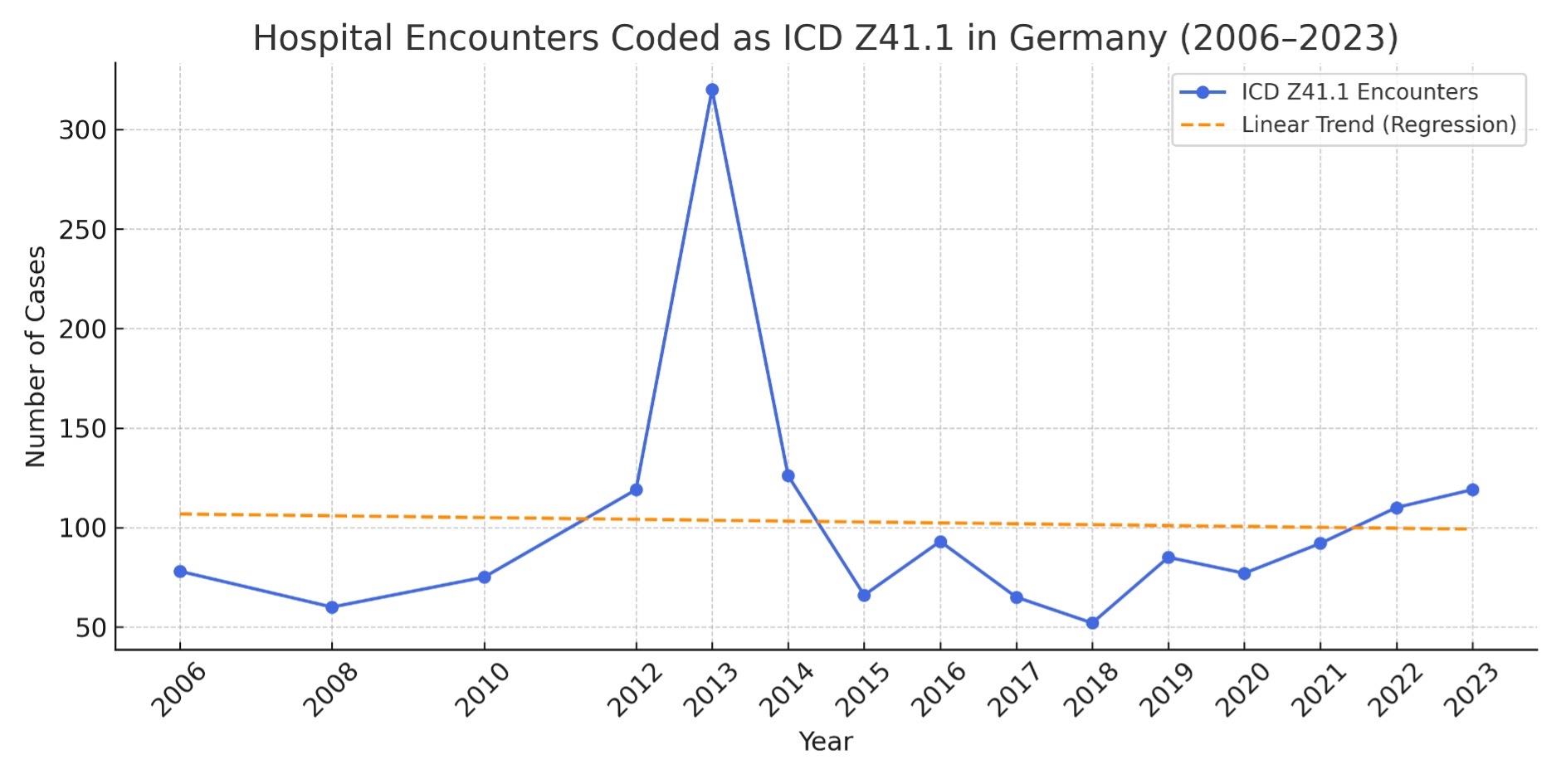

Supplement: Supplementary file 1 [file Image_1.jpeg]

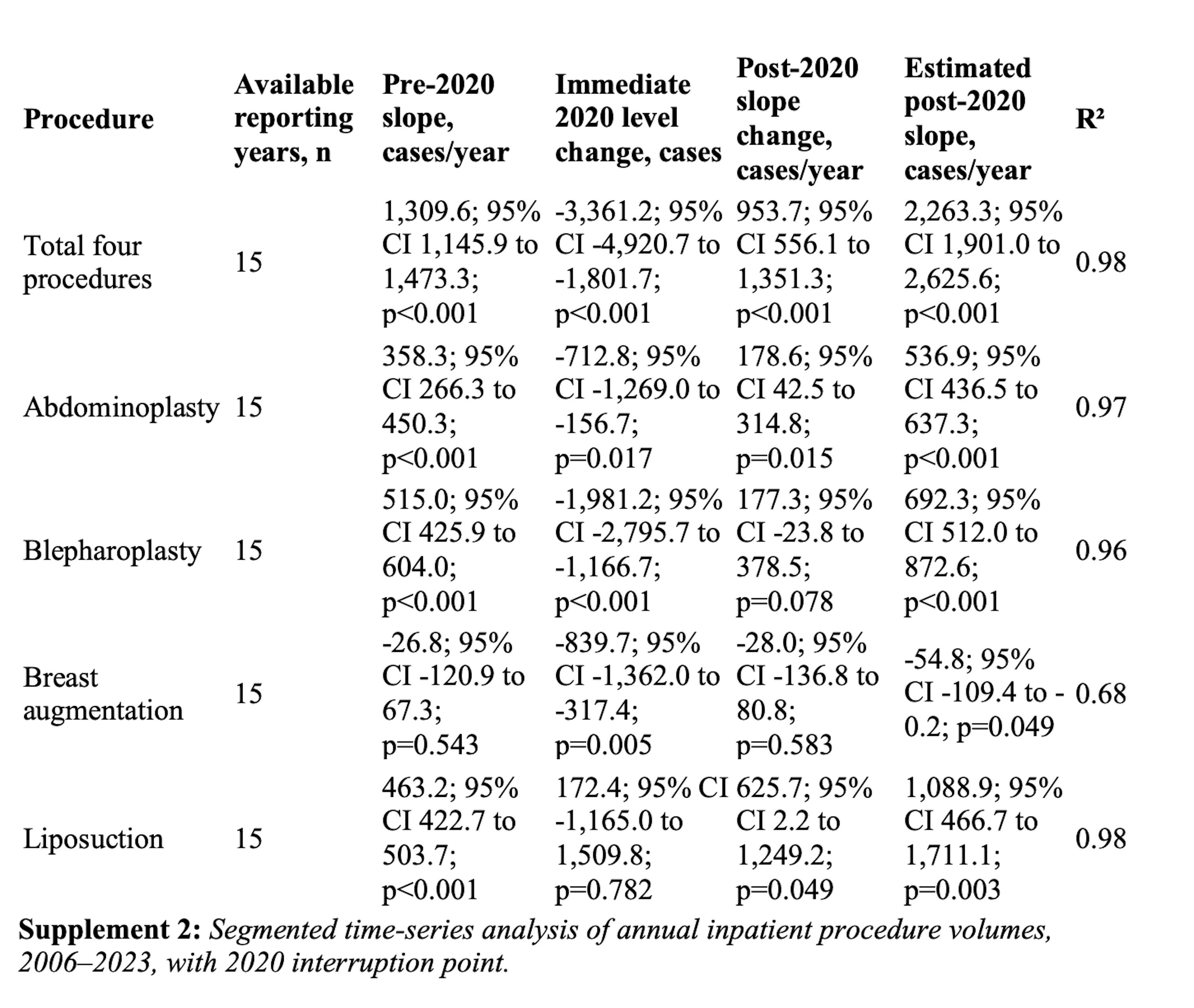

Supplement: Supplementary file 2 [file Image_2.jpeg]
